# The mitochondrial DNA constitution shaping T-cell immunity in patients with rectal cancer at high risk of metastatic progression

**DOI:** 10.1007/s12094-021-02756-w

**Published:** 2021-12-27

**Authors:** P. A. Bousquet, S. Meltzer, A. J. Fuglestad, T. Lüders, Y. Esbensen, H. V. Juul, C. Johansen, L. G. Lyckander, T. Bjørnetrø, E. M. Inderberg, C. Kersten, K. R. Redalen, A. H. Ree

**Affiliations:** 1 Department of Oncology, Akershus University Hospital, Lorenskog, Norway; 2 Institute of Clinical Medicine, University of Oslo, Oslo, Norway; 3 Centre for Cancer Treatment, Sørlandet Hospital, Kristiansand, Norway; 4 Department of Clinical Molecular Biology, Akershus University Hospital, Lorenskog, Norway; 5 Department of Cellular Therapy, Oslo University Hospital, Oslo, Norway; 6 Department of Pathology, Akershus University Hospital, Lorenskog, Norway; 7 Department of Physics, Norwegian University of Science and Technology, Trondheim, Norway

**Keywords:** CD4, Colorectal cancer, Immune cells, Metastasis, Mitochondrial DNA

## Abstract

**Purpose:**

A significant percentage of colorectal cancer patients proceeds to metastatic disease. We hypothesised that mitochondrial DNA (mtDNA) polymorphisms, generated by the high mtDNA mutation rate of energy-demanding clonal immune cell expansions and assessable in peripheral blood, reflect how efficiently systemic immunity impedes metastasis.

**Patients and methods:**

We studied 44 rectal cancer patients from a population-based prospective biomarker study, given curative-intent neoadjuvant radiation and radical surgery for high-risk tumour stage and followed for metastatic failure. Blood specimens were sampled at the time of diagnosis and analysed for the full-length mtDNA sequence, composition of immune cell subpopulations and damaged serum mtDNA.

**Results:**

Whole blood total mtDNA variant number above the median value for the study cohort, coexisting with an mtDNA non-H haplogroup, was representative for the mtDNA of circulating immune cells and associated with low risk of a metastatic event. Abundant mtDNA variants correlated with proliferating helper T cells and cytotoxic effector T cells in the circulation. Patients without metastatic progression had high relative levels of circulating tumour-targeting effector T cells and, of note, the naïve (LAG-3^+^) helper T-cell population, with the proportion of LAG-3^+^ cells inversely correlating with cell-free damaged mtDNA in serum known to cause antagonising inflammation.

**Conclusion:**

Numerous mtDNA polymorphisms in peripheral blood reflected clonal expansion of circulating helper and cytotoxic T-cell populations in patients without metastatic failure. The statistical associations suggested that patient’s constitutional mtDNA manifests the helper T-cell capacity to mount immunity that controls metastatic susceptibility.

**Trial registration:**

ClinicalTrials.gov NCT01816607; registration date: 22 March 2013.

**Supplementary Information:**

The online version contains supplementary material available at 10.1007/s12094-021-02756-w.

## Introduction

Colorectal cancer (CRC) is a heterogeneous disease of high biological complexity [1, 2]. A significant percentage of CRC patients proceeds to metastatic disease, which remains the cause of severe morbidity and dismal survival. The propensity of systemic CRC dissemination comprises the interrelation between the cancer and the immune system. Tumour-defeating immunity entails the activation of cytotoxic lymphocytes. But protective mechanisms against auto-immunity impede the immune surveillance and create immune tolerance that is manifested within the tumour microenvironment (TME) [3] and frequently exerted as detrimental systemic inflammation [4]. The state of the immune cell metabolism may be a decisive factor in the balance between surveillance, tolerance and inflammation, particularly reflected in the enormous energy demand of activated T cells when exponentially proliferating to mount efficient immunity [5, 6]. Yet, the understanding of how T cells incite tumour immunity is limited by the scarce information on the distribution of individual T-cell clones in peripheral blood [7].

Compelling discoveries in mouse models have brought new insights into the role of metabolic reprogramming during clonal T-cell activation and differentiation, a feature exploited by the immunologically tolerant TME where the mitochondrial biogenesis of T cells is driven in the direction of dysfunctional tumour-defeating activity [8–11]. For example, metabolic adaptation is crucial for the specific accommodation of the immune-suppressive regulatory T cells to the hostile TME conditions [12, 13]. In patient samples, single-cell RNA and T-cell receptor sequencing has indicated that clonal replacement of T cells into the tumour, rather than expansion from pre-existing clones within, is the predominant mechanism of response to therapeutic immune checkpoint blockade (ICB) [14, 15]. Moreover, expanded tumour-residing T-cell clones have been detected as highly expanded in the peripheral blood too, suggesting that the TME clones are recruited from a distinct T-cell repertoire in the systemic circulation [7]. It is tempting to speculate if circulating clones may stand proxy for tumour-specific T cells, analogous to the case recently reported for patients with advanced-stage gastrointestinal cancer responding to immunogenic chemotherapy and ICB [16], and inform on the state of immune surveillance versus tolerance [15].

The mitochondrial genome is a 16569-base circular molecule encoding subunits of the enzyme complexes that drive oxidative phosphorylation. Mammalian cells harbour a dynamic mitochondrial network with numerous mitochondrial DNA (mtDNA) copies that can affect the clinical phenotype [17, 18]. Because the mutation frequency of replicating mtDNA is high, mutant mtDNA copies are often mixed with wild-type copies in the cell. The mtDNA polymorphisms may alter mitochondrial function, particularly in tissues that are highly dependent on the metabolism [18] such as activated immune cells. Nevertheless, if a mutation is pathogenic, the cell can often tolerate a certain proportion of the mtDNA variant before the biochemical threshold is exceeded with resulting metabolic defects [18].

We hypothesised that circulating immune cells display mtDNA polymorphisms resulting from the high mtDNA mutation rate of an energy-demanding clonal activation, which reflect how efficiently patients generate systemic immunity against CRC metastasis. The hypothesis also covered the concept of damaged mtDNA (i.e., mtDNA with structural modifications that are unrelated to mutations), which on relocation into the cell’s cytosol induces a cytokine response [19] and further, in the circulation after cellular injury, contributes to elicit a systemic inflammatory response [20]. The release of damaged mtDNA from dysfunctional immune cells in the TME or circulation is pivotal in the systemic inflammatory response [21].

We studied patients with rectal adenocarcinoma enrolled onto a prospective population-based biomarker study, who were planned for curative-intent radiation therapy before definitive surgery, yet at high risk of metastatic progression beyond the pelvic cavity. The setting of non-metastatic but high-risk tumour stage is ideal to study how the immune system controls metastatic dissemination since a certain percentage of patients will proceed to metastatic disease even after curative-intent therapy. The patients had full-length mtDNA sequencing of the whole blood (WB), the composition of peripheral blood mononuclear cell (PBMC) subpopulations analysed and damaged mtDNA in serum assessed in the various blood compartments sampled at the time of diagnosis. Metastatic events were recorded up to 60 months of follow-up after completion of the multimodal treatment.

## Materials and methods

### Patients and procedures

The study was conducted at Akershus University Hospital. Details of the eligibility criteria, diagnostic workup, neoadjuvant therapy and local treatment response (histologic ypTN stage and tumour regression grade) are given in Supplementary methods. The 44 patients reported here were enrolled between 28 October 2013 and 14 November 2017 and treated according to the prevailing national guidelines with neoadjuvant radiation therapy and radical pelvic surgery. Of note, two patients refused surgery due to personal opinions. Patients were followed with regular clinical and computed tomography examinations for 5 years after the completion of the multimodal treatment to record metastatic events in distant organs beyond the pelvic cavity (defining distant metastasis-free survival; DMFS), with median follow-up of 38 (minimum 2, maximum 60) months at censoring on 2 January 2020.

### Patient samples

WB samples were collected by venipuncture in PAXgene RNA tubes (PreAnalytiX) at the time of diagnosis, for DNA extraction undertaken after median 45 (minimum 16, maximum 66) months of storage at − 80 °C. Total DNA was extracted using the DNeasy Blood & Tissue Kit (Qiagen) and the DNA was quantified using the Qubit fluorometer 2.0 (Thermo Fisher Scientific) in combination with the Qubit dsDNA HS Assay Kit (Thermo Fisher Scientific). PBMC specimens were prepared from 6 to 8 mL of the WB, as detailed in Supplementary methods, and immediately frozen in − 150 °C. For serum preparation, WB was drawn in plain tubes with no additives for centrifugation to separate serum, which was left on ice for no more than 1 h before storage at − 80 °C.

### Healthy blood donor samples

PBMC preparations, by the Lymphoprep density gradient medium (Stem Cell Technologies), from healthy blood donors at Oslo University Hospital were allowed for research purposes according to each individual’s written informed consent. Anonymised samples without any clinical or biological information were made available for this study.

### Library preparation and sequencing

The mtDNA was initially amplified in two PCRs to generate two long fragments spanning the complete human mitochondrial genome (16569 bases long). The process of library construction occurred following the suggested Illumina protocol [22] but specifically, a temperature gradient (51–68 °C) was used during the first PCR amplification. The DNA was subsequently purified using gel electrophoresis and bands representing the mtDNA amplicons (9.1 and 11.2 kilobases of length) were cut out from the gel. Extraction and quantification of the mtDNA were performed using QIAEX II Gel Extraction Kit (Qiagen) and Qubit dsDNA HS Assay Kit. The mitochondrial genomes were sequenced using the Nextera XT DNA Library Preparation Kit, Nextera XT Index Kit and a MiSeq Benchtop Sequencer (all Illumina). The full mtDNA was successfully sequenced at a minimum coverage of 2000×.

### Sequence analysis

The sequence data were mapped to the revised Cambridge Reference Sequence (GenBank ID: NC_012920.1) [23, 24] using the MiSeq Reporter built-in software version 2.6 (Illumina). This software applies a Burrows-Wheeler Aligner [25] and the Genome Analysis ToolKit [26] for variant calling of single nucleotide polymorphisms and short indels. It produces graphical representations of average and coverage quality scores along with tabular output of the variant calls. The analyses applied default settings for quality of base calls, detection threshold and analysis threshold. All variant call format files were imported into two software suites for variant interpretation. One was HaploGrep2 (haplogrep.i-med.ac.at) that determines mtDNA haplogroup classification. It is based on pre-calculated phylogenetic weights that correspond to the occurrence per position in PhyloTree_mt_ [27], reflecting the mutational stability of a given variant [28, 29]. The other was the Ensembl Variant Effect Predictor tool that performs analysis of functional effects of DNA mutations [30]. Twelve of the study patients were randomly selected for comparison of mtDNA variants in WB and PBMC samples.

### High-dimensional single-cell mass cytometry

PBMC preparations were available from 32 of the rectal cancer patients and ten healthy blood donors for this analysis. The procedures are detailed in Supplementary methods. The mass cytometry data were analysed by the online Cytobank software (cytobank.org). For each PBMC sample, the gating was done by the analyst blinded for other study data. The gating strategy applied EQ-140 versus time to exclude calibration beads, event length versus Ir-191 to gate singlets and cisplatin versus CD45-89Y to select live cells for further analysis. Specifically, subsets of T cells (CD3^+^CD4^+^ including CD3^+^CD4^+^CD127^lo/–^CD25^hi^ and CD3^+^CD8^+^ with subtypes), naïve (IgD^+^CD27^–^) and memory (CD20^+^CD27^+^) B cells and monocytes (CD33^+^CD11b^+^CD14^+^) were identified for quantification.

### Quantification of mtDNA damage

The assay relies on the ability of a structural modification within a 4-base site (TCGA, which did not exhibit polymorphisms in any of the patient samples) on the template DNA to inhibit restriction enzyme cleavage [31]. We have previously published the detailed procedure for digital PCR analysis of serum mtDNA damage [32].

### Analysis of serum cytokines

The simultaneous analysis of 84 serum proteins, including interleukin (IL)-6 and interferon (IFN)-γ, was undertaken with a customized Luminex Multiplex Assays (R&D Systems). To enable comparability among and within the range of serum levels of analytes, which were measured with the required number of assay batches, all levels were normalized to the mean value of each analyte in each batch before use in statistical calculations.

### Statistical analysis

Analyses were preformed using IBM SPSS Statistics for Mac version 27 and GraphPad Prism version 9.3.0. The Shapiro–Wilk test was applied to examine if the variables were normally distributed. Correlations were calculated using Pearson correlation analysis (when the data set had parametric distribution) or Spearman correlation analysis (if the data set contained any non-parametric distributions). Scatter graphs for significant correlations are shown in Supplementary Fig. S1. Differences between groups were compared by Student’s *t* test or one-way analysis of variance, as appropriate. Associations between various forms of mtDNA variant number, the PBMC phenotype or serum cytokines and DMFS were determined from univariable Cox proportional hazard models. Any associations between DMFS and the WB-mtDNA variant number relative to other potentially prognostic parameters was determined from a multivariable Cox proportional hazard model with forward conditional selection of given patient and disease characteristics. The results were presented as hazard ratio with 95% confidence interval. Crude differences in DMFS were assessed by the log-rank test and visualised by the Kaplan–Meier method. All tests were two-sided and *p* < 0.05 was considered statistically significant.

## Results

### Patient and disease characteristics

The 44 study cases had typical patient and baseline tumour characteristics of a rectal cancer population (Supplementary Table S1). All patients had microsatellite-stable tumour. The neoadjuvant radiation resulted in a high rate (43.2%) of near-complete or complete local treatment response (histologic tumour regression grade 1).

For each of the 12 randomly selected patients (Supplementary Fig. S2), the PBMC specimen revealed essentially identical mtDNA variants as the WB counterpart but had some additional variants (*r* = 0.939, *p* < 0.001; by Pearson correlation test), which potentially reflected artificial DNA aberrations introduced during the PBMC preparation. We considered each patient’s WB-mtDNA to be representative of the mitochondrial genome in circulating immune cells.

### WB-mtDNA variant number, haplogroups and patient outcome

**Fig. 1:**
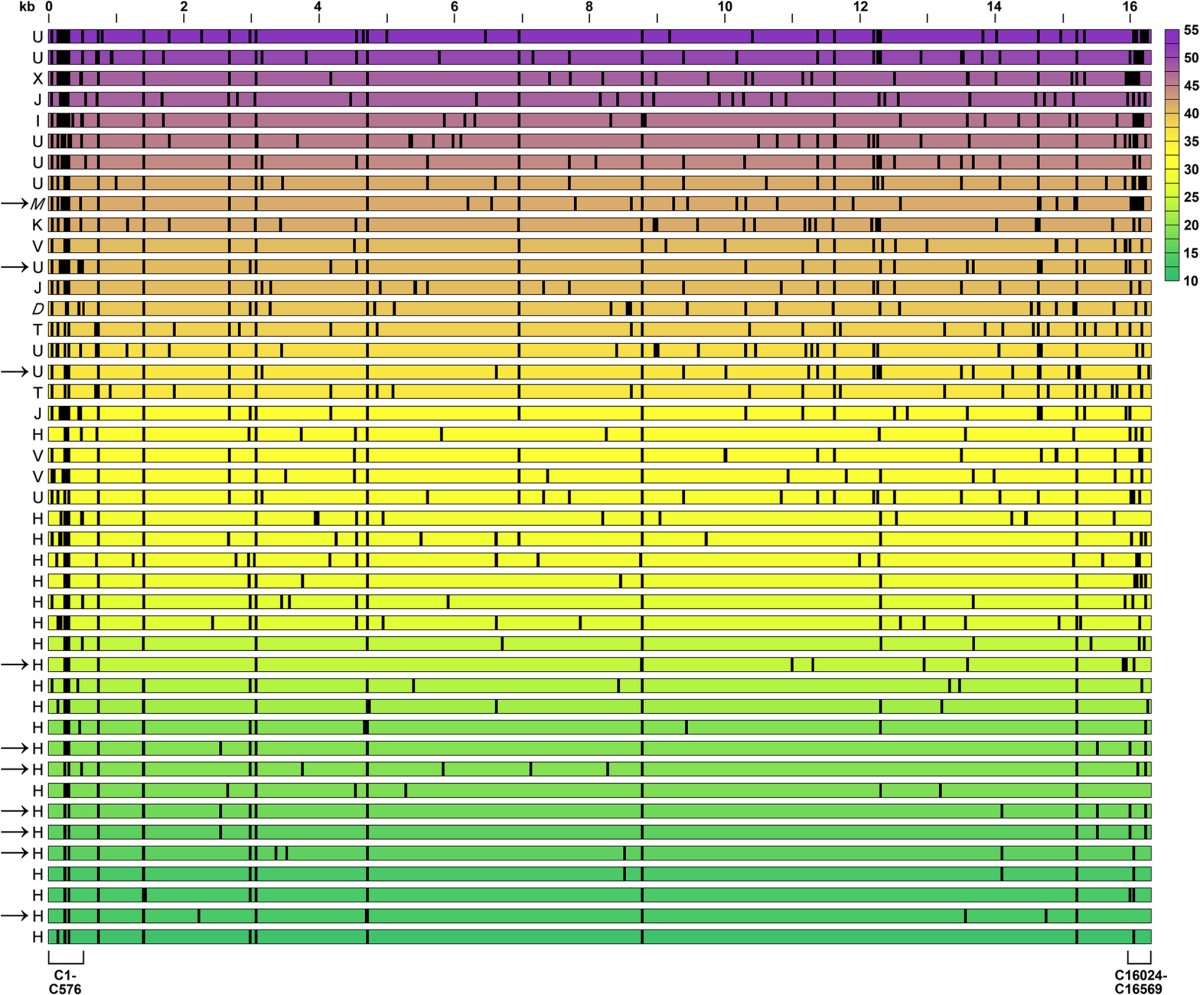
Mitochondrial DNA (mtDNA) variant number in whole blood from 44 high-risk rectal cancer patients. Each row represents one individual patient with the haplogroup shown to the left (the non-European M and D haplogroups in italic). The positions across the entire ~ 16.6 kilobase (kb) mitochondrial genome are indicated on the top of the panel. The control (C) region’s base sites 1–576 and 16024–16569 are specified below the panel. Arrows mark patients who had metastatic progression during the follow-up after completion of the curative-intent therapy; one of the metastatic haplogroup U cases was a patient who had declined primary tumour surgery (i.e., refused the full treatment). Each vertical line indicates the position of an mtDNA base with at least one variant other than the reference, as called by the curated reference genome (mitomap.org). The total number of base variants in each case is indicated by colour (scale to the right)

The WB-mtDNA total variant number (TVN) was strikingly different across the 44 study cases with median value of 30.5 (minimum 13, maximum 53; Fig. 1) but without correlation to patient’s age, body mass index or metformin use, which may affect mitochondrial function, or to female or male sex, the disease extension at presentation or the local tumour response to the neoadjuvant therapy (Supplementary Table S1). We observed a clear arrangement of mtDNA haplogroups in this population-based rectal cancer cohort from Norway. The 22 patients harbouring the common H haplogroup [33], the most recent addition to European mtDNAs [34, 35], had TVN of 29 or below (except one case with TVN of 32), while the 20 patients with other European haplogroups and the two Asian haplogroup cases had TVN above the median value (of whom all but four had TVN of 36 or higher; Fig. 1). The fraction of missense or frameshift mutations was median 0.41 (minimum 0.16, maximum 0.73) for non-H haplogroup cases, while the haplogroup H cases had a significantly higher fraction of median 0.58 (minimum 0.33, maximum 0.86) of such variants that can confer altered clinical phenotypes (*p* < 0.001). Of ten patients with metastatic progression during follow-up, seven belonged to haplogroup H.

Accordingly, the higher WB-mtDNA TVN, the longer DMFS, an association that was lost when each patient’s haplogroup-specific variants were omitted. Better DMFS also pertained to higher variant number of the mtDNA hypervariable region (HVR) 2–3 but was not associated with the number of variants of the mtDNA coding region. After omission of the ancestral variants, however, an association was also found between favourable DMFS and high coding region count (Table 1). These findings suggest that abundant private polymorphisms reflect an advantageous circulating immune cell function in high-risk rectal cancer, and that the polymorphisms of the mtDNA sequence most receptive to base substitutions (HVR2-3) contributed with the lowest risk for metastatic failure. In a multivariable model that in a forward conditional selection manner included other potentially prognostic patient and disease characteristics (from Supplementary Table S1), only the mtDNA variant number remained prognostic for DMFS (Supplementary Table S2).

**Table 1:** Associations between WB-mtDNA variant numbers and DMFS

	*n*	HR (95% CI)	*p*
TVN	44	0.935 (0.878–0.996)	0.037
TVN without haplogroup-specific variants	44	1.076 (0.862–1.344)	0.515
TVN without AV	44	0.918 (0.849–0.992)	0.031
CRVN	44	0.912 (0.830–1.002)	0.055
CRVN without AV	44	0.875 (0.768–0.997)	0.046
HVR1 (C16024-C16569) variant number	44	0.998 (0.807–1.233)	0.982
HVR1 variant number without AV	44	1.002 (0.801–1.254)	0.983
HVR2-3 (C1-C576) variant number	44	0.692 (0.517–0.928)	0.014
HVR2-3 variant number without AV	44	0.704 (0.520–0.953)	0.023
Total AV number	44	0.845 (0.682–1.047)	0.124

HR below 1 indicates favourable DMFS with higher number of mtDNA base variants, from Cox proportional hazard models

*AV* ancestral variants, *C* control region, *CI* confidence interval, *CRVN* coding region variant number, *DMFS* distant metastasis-free survival, *HR* hazard ratio, *HVR* hypervariable region (the C region’s base sites within the HVRs are given in brackets), *TVN* total variant number, *WB-mtDNA* whole blood mitochondrial DNA

### A specific *MT-RNR2* variant and patient outcome

Next, we examined whether the presence of single nucleotide deletion polymorphisms at specific mtDNA sites might inform on the susceptibility to disease dissemination. The state of all mtDNA molecules within a cell being identical is termed homoplasmy. The state of a mixture of wild-type and mutant variants (at a specific mtDNA site) is termed heteroplasmy. The patients’ WB-mtDNA sequence data revealed a median of 2 (minimum 1, maximum 6) deletions, which we inspected manually. Of these, the 3105 site of the *MT-RNR2* gene was either wild-type homoplasmic or had a high level of heteroplasmy. Specifically, as illustrated in Supplementary Fig. S3, 24 patients had homoplasmy and 20 showed heteroplasmy of 0.964–0.995 for the AC>A deletion variant. Of the ten recorded metastatic events, nine belonged to patients with a homoplasmic site, while the single event among heteroplasmic cases appeared in a patient who had declined primary tumour surgery (i.e., refused the full curative-intent therapy), resulting in significantly different DMFS for the opposite variant groups (*p* = 0.014; by log-rank test). The haplogroup H cases were equally split in number between the groups. Among haplogroup H patients with the unfavourable *MT-RNR2* variant, 58.3% progressed to metastatic disease. Altogether, the findings suggest that the absence of 3105 AC>A deletion in *MT-RNR2*, which encodes one of the two ribosomal subunits and is, thus, fundamental for the intact mitochondrial function, in a haplogroup H genome is statistically associated with insufficient immune cell activity and metastatic failure in high-risk patients.

### mtDNA variant number and circulating T-cell populations

We investigated whether the WB-mtDNA diversity might relate to any particular circulating immune cell population and were able to undertake high-dimensional single-cell mass cytometry of PBMC samples from 32 (72.7%) of the study participants, of whom seven were among the total of ten cases with metastatic events during follow-up. Plasma membrane markers were analysed in all of the samples, while half the samples (none with metastatic events) were available for analysis of PBMC subset-specific and additional intracellular markers following short in vitro-stimulation of cytotoxicity and cytokine production. For comparison, we also analysed PBMC specimens from ten healthy individuals (blood donors) with median mtDNA TVN of 24.5 (minimum 7, maximum 44), of whom all of the five individuals with the H haplogroup were below the median (Supplementary Fig. S4).

Concerning the PBMC samples analysed for both plasma membrane and intracellular markers (Table 2), two cell subsets appeared to be of interest; CD3^+^CD4^+^ (helper T cells) and CD3^+^CD8^+^ (effector T cells). The TVN of both WB-mtDNA in the 16 patients and PBMC-mtDNA in the ten healthy individuals showed clear correlation with the number of proliferating (Ki67^+^) helper T cells, being a small CD3^+^CD4^+^ fraction [for the patients: mean of 1.39%, standard deviation (SD) 1.33%]. Pertaining to patient samples only, the TVN also correlated with the percentage of effector T cells positive for granzyme B (mean of 41.4%, SD 25.7%) and perforin (mean of 18.5%, SD 14.6%), indicating functional activity [36].

**Table 2:** PBMC phenotypes and correlations with mtDNA TVN

	Subset percentage of total PBMC	
	CD3^+^CD4^+^	CD3^+^CD8^+^
Patients (*n* = 16)	33.9 (8.03)	23.1 (9.38)
Donors (*n* = 10)	44.8 (7.01)	21.5 (3.43)

	Subtype percentage of PBMC subset		
	CD3^+^CD4^+^ Ki67^+^	CD3^+^CD8^+^ Granzyme B^+^	CD3^+^CD8^+^ Perforin^+^
Patients (*n* = 16)	1.39 (1.33)	41.4 (25.7)	18.5 (14.6)
Donors (*n* = 10)	1.49 (0.68)^a^	18.8 (12.3)	10.7 (6.38)

	Correlation between mtDNA TVN and the PBMC subtype number^b^					
	rho	*p*	rho	*p*	rho	*p*
Patients (*n* = 16)	0.734	0.001	0.534	0.033	0.590	0.016
Donors (*n* = 10)	0.687	0.033				

PBMC numbers are given by mean (standard deviation)

*mtDNA* mitochondrial DNA, *PBMC* peripheral blood mononuclear cell, *TVN* total variant number

^a^Not normally distributed

^b^Statistically significant correlations with mtDNA TVN are shown, by Spearman correlation test

### mtDNA variant number and the shaping of T-cell help

Concerning the 32 patient PBMC samples analysed for plasma membrane markers solely, WB-mtDNA TVN was weakly correlated only with the CD3^+^CD4^+^ cell fraction positive for the lymphocyte activation gene (LAG)-3 (*r* = 0.378, *p* = 0.033; by Pearson correlation test), constituting a tiny fraction (mean of 0.14%, SD 0.13%) of the circulating helper T cells. No others of the analysed T cell populations were correlated with mtDNA variant numbers, nor were the circulating populations of naïve and memory B cells or monocytes. Nevertheless (Table 3), the more LAG-3-expressing CD3^+^CD4^+^ cells, representing a naïve CD3^+^CD4^+^ population from which new helper T cells can be rapidly recruited [37], the better DMFS, with remarkably low risk for metastatic failure in patients with LAG-3^+^ cell counts in the upper range. A similar observation was recently reported in advanced-stage gastric cancer [38]. Favourable DMFS also pertained to higher fraction of circulating CD3^+^CD8^+^ cells positive for CXCR4 (mean of 81.5%, SD 11.4%), a chemokine receptor that promotes their infiltration into tumour sites [37, 39]. The number of circulating monocytes (CD33^+^CD11b^+^CD14^+^) seemed to be neutral with regard to DMFS and the naïve and memory B-cell populations (IgD^+^CD27^–^ and CD20^+^CD27^+^) as well as the fraction of immune-suppressive regulatory T cells (CD3^+^CD4^+^CD127^lo/–^CD25^hi^) were statistically unrelated. The composition of circulating immune cell phenotypes in a representative patient without metastatic failure is visualised in Supplementary Fig. S5 along with cell population frequencies in all of the patient samples.

**Table 3:** PBMC phenotypes, inflammation and associations with DMFS

	*n*	HR (95% CI)	*p*
CD3^+^CD4^+^	32	1.003 (0.939–1.071)	0.938
CD3^+^CD4^+^LAG-3^+^	32	< 0.001 (< 0.001–0.378)	0.032
CD3^+^CD4^+^CD127^lo/−^CD25^hi^	32	1.000 (0.997–1.004)	0.793
CD3^+^CD8^+^	32	0.987 (0.902–1.080)	0.779
CD3^+^CD8^+^CXCR4^+^	32	0.915 (0.857–0.978)	0.009
IgD^+^CD27^−^	32	0.960 (0.914–1.007)	0.096
CD20^+^CD27^+^	32	1.045 (0.988–1.106)	0.127
CD33^+^CD11b^+^CD14^+^	32	1.000 (1.000–1.000)	0.023
IL-6	42	1.460 (1.069–1.993)	0.017
IFN-γ	25	1.000 (0.999–1.001)	0.868

HR below 1 indicates favourable DMFS with higher PBMC population count or lower serum cytokine value, from Cox proportional hazard models; patients’ cytokine values were normalised to the mean value of each analysis assay batch for use in the model

*CI* confidence interval, *DMFS* distant metastasis-free survival, *HR* hazard ratio, *IFN* interferon, *IL* interleukin, *LAG* lymphocyte activation gene, *PBMC* peripheral blood mononuclear cell

In the blood donors, no correlation was found between PBMC-mtDNA TVN and immune cell populations CD3^+^CD4^+^LAG-3^+^ or CD3^+^CD8^+^CXCR4^+^. Although proof of a mechanistic link is missing, the PBMC data supported the notion that high mtDNA TVN of circulating immune cells reflects the expansion of helper T cells and, in patients, the resulting effector T population endowed with tumour-infiltrating and activated cytotoxic properties, which may restrain the metastatic propensity of high-risk rectal cancer.

### Systemic inflammation

On entering the circulation after cellular stress, cell-free mtDNA may trigger detrimental pro-inflammatory responses by inducing factors such as IL-6 [21], which maintains immune tolerance through its ability to attenuate intratumoural activity of helper and cytotoxic T cells [40, 41] and is regarded a main mediator and representative cytokine of cancer inflammation [41, 42]. Besides, mtDNA release triggers type I IFN responses [21]. In the study cases, no association was found between damaged mtDNA in serum (mean of 23.5%, SD 6.0% of the total cell-free mtDNA) and IL-6 [median value of 2.72 (minimum 0, maximum 75.0) pg/mL]. Yet, elevated IL-6 was associated with unfavourable DMFS (Table 3), in accordance with observations in established metastatic CRC [43]. And reflecting the association between poor DMFS and low WB-mtDNA variant counts (Table 1), the IL-6 level was inversely (though not strongly) correlated to the HVR2-3 variant number in circulating immune cells (Supplementary Table S3). Serum IFN-γ [median value of 1.50 (minimum 0.43, maximum 3.34) ng/mL] was not associated with DMFS (Table 3). Finally, following the case for DMFS, the number of circulating LAG-3-expressing CD3^+^CD4^+^ cells was inversely correlated with the damaged serum mtDNA (Table 4).

**Table 4:** PBMC phenotypes and correlations with damaged serum mtDNA

	*n*	rho	*p*
CD3^+^CD4^+^	32	− 0.199	0.275
CD3^+^CD4^+^LAG-3^+^	32	− 0.703	< 0.001
CD3^+^CD4^+^CD127^lo/−^CD25^hi^	32	− 0.245	0.176
CD3^+^CD8^+^	32	− 0.054	0.769
CD3^+^CD8^+^CXCR4^+^	32	− 0.286	0.112
IgD^+^CD27^−^	32	− 0.228	0.209
CD20^+^CD27^+^	32	− 0.082	0.657
CD33^+^CD11b^+^CD14^+^	32	− 0.328	0.067

Calculated by Spearman correlation test

*LAG* lymphocyte activation gene, *mtDNA* mitochondrial DNA, *PBMC* peripheral blood mononuclear cell

The present report is based on statistical associations, demanding cautious biological interpretations. But collectively, if the blood mtDNA status reflects the balance of advantageous immunity versus adverse inflammation in high-risk rectal cancer, tumour-targeting cytotoxic immune cells (CD3^+^CD8^+^CXCR4^+^) that are dependent on helper cells (CD3^+^CD4^+^Ki67^+^) sustained by a recruitment population (CD3^+^CD4^+^LAG-3^+^) [36, 37, 44] may hold fundamental functions in this context.

## Discussion

Since a deficient immune cell surveillance repertoire is a result of an insufficient clonal expansion of tumour-defeating immune cells, we hypothesised that the energy-demanding antitumour-proficient state may generate mutations in the rapidly replicating mtDNA that are detectable in a WB specimen at the time of cancer diagnosis. We observed that patients with rectal cancer at high risk of systemic dissemination who presented a high number of WB-mtDNA polymorphisms, being representative for the mtDNA of circulating immune cells, had favourable survival without development of metastatic disease after neoadjuvant radiation enabling complete resection of the residual primary tumour. Numerous mtDNA variants correlated with more of actively proliferating helper T cells and effector T cells with activated cytotoxic properties in the circulation. High relative levels of tumour-targeting effector T cells and the naïve (LAG-3^+^) helper T population protected patients from metastatic progression, with the proportion of LAG-3^+^ cells inversely correlating with the serum content of antagonising pro-inflammatory damaged mtDNA.

In further support of our assumption, a base deletion in a ribosomal subunit gene (*MT-RNR2*) pivotal for the mitochondrial protein synthesis was associated with the likelihood of inferior outcome in this population of rectal cancer patients prone to metastatic progression. The compelling discovery that patients with total mtDNA variant number below the median for the study cohort invariably carried the common H haplogroup, which also was the case among the healthy blood donors, agrees with the notion that haplogroup H replacement mutations enabled migrating humans in ancient times the metabolic adaptation to colder European climates [45] by yielding the cellular capacity to meet a high energy demand to that of generating heat instead [46]. The findings in patients with sepsis, the clinical syndrome of multiple-organ failure after microbial invasion, that the H haplogroup was a strong independent predictor of increased chance of survival [47, 48] are consistent with a constraint to mount excessive and deleterious immune responses. This may have caused survival advantage for ancestors of the present populations of very-high Human Development Index regions. Almost 60% of the participants in our study bearing the combination of haplogroup H and the unfavourable variant of the ribosomal subunit gene experienced metastatic progression. One might speculate if functional haplogroup H polymorphisms in combination with the given *MT-RNR2* variant lead to synergistic immune cell vulnerabilities that favour the control of systemic infection but impede the control of systemic cancer dissemination.

The effector T-cell phenotype was equally prevalent in the circulation of the healthy individuals and rectal cancer patients but unsurprisingly with much higher cytotoxic activity (granzyme B and perforin expression, correlating with the WB-mtDNA TVN) in the patients. The finding that much of the circulating CD3^+^CD8^+^ subset was positive for the chemokine receptor CXCR4 in patients with long DMFS is in line with data from a mouse model, in which CXCR4 expression bestowed the cytotoxic T cells the function to reach the tumour target tissue [39]. Interestingly, this effector program, also including induced granzyme B and perforin expression and altered metabolic processes, was acquired after specific CD3^+^CD4^+^ help [39].

The strong correlation between WB-mtDNA TVN and the number of circulating helper T cells under active proliferation in both the blood donors and rectal cancer patients might mean that an individual’s proportion of CD3^+^CD4^+^Ki67^+^ cells directly reflects the inherited capacity of clonal immune cell expansion. In mice, the homeostatic expansion of naïve helper T cells was shown to be metabolically regulated through the coinhibitory immune checkpoint receptor LAG-3, ensuring firmly controlled CD3^+^CD4^+^ cell activation [37]. In our study, upper range counts of the diminutive CD3^+^CD4^+^LAG-3^+^ population correlated positively with survival without metastasis and inversely with pro-inflammatory mtDNA in serum. This novel finding argues a critical function of naïve CD3^+^CD4^+^ cells in the balance of advantageous antitumour immunity versus adverse systemic inflammation and thus, for the outcome of high-risk rectal cancer. However, a biomarker study based on statistical associations in patients, such as ours, does not provide direct proof of any mechanistic link between the WB-mtDNA variant number and the activity of tumour-defeating immune cells.

The present study population validated our previous observations in high-risk rectal cancer patients of strong association between cell-free damaged mtDNA in the circulation and systemic inflammatory factors [32]. In the present investigation, we chose to analyse serum IL-6 because, in CRC patients, IL-6 has been shown directly linked to the clinical presentation of the systemic inflammatory response, such as skeletal muscle wasting under metabolic malfunctioning [49]. We confirmed that elevated IL-6 was predictive of unfavourable DMFS but seemed not to be an effector of the cell-free damaged mtDNA.

CRC is one of the most common malignancies worldwide with a particularly high incidence in very-high Human Development Index regions [50]. Approximately a third of rectal cancer patients given neoadjuvant radiation experience metastatic failure [51, 52], which is the result of distant organ colonisation by surviving clonogenic tumour cells. The constitutional immunity of the patient may be of note in this context. Because the dynamic metabolism during activation of quiescent helper T cells and the resulting cytotoxic effector program that can eliminate clonogenic tumour cells have been explored predominantly in mouse models, proving the same responses may occur in patients can become the next step in clinical cancer immunology. With the purpose of improving diagnostic precision and treatment personalisation, it is feasible to undertake mtDNA sequencing of peripheral blood to assess the TVN. Even determining the mtDNA haplogroup type may suffice. The statistical associations presented in this study suggest that a patient’s constitutional mtDNA manifests the helper T-cell capacity to mount immunity that controls metastatic susceptibility in CRC.

## Supplementary Information

Below is the link to the electronic supplementary material.

Supplementary file1 (DOCX 750 kb)

## Data Availability

Sequence data have been deposited at the European Genome-phenome Archive (EGA), which is hosted by the EBI and the CRG, under accession number EGAS00001005540. Further information about EGA can be found on https://ega-archive.org. Other datasets used and analysed during the current study are available from the corresponding author on reasonable request and in accordance with the General Data Protection Regulation of the European Union.

## References

[1] Dienstmann R, Vermeulen L, Guinney J (2017). Consensus molecular subtypes and the evolution of precision medicine in colorectal cancer. Nat Rev Cancer.

[2] Ros J, Baraibar I, Martini G (2021). The evolving role of consensus molecular subtypes: a step beyond inpatient selection for treatment of colorectal cancer. Curr Treat Options Oncol.

[3] Katlinski KV, Gui J, Katlinskaya YV (2017). Inactivation of interferon receptor promotes the establishment of immune privileged tumor microenvironment. Cancer Cell.

[4] Pennel KAF, Park JH, McMillan DC (2019). Signal interaction between the tumour and inflammatory cells in patients with gastrointestinal cancer: implications for treatment. Cell Signal.

[5] Jung J, Zeng H, Horng T (2019). Metabolism as a guiding force for immunity. Nat Cell Biol.

[6] Teijeira A, Garasa S, Etxeberria I (2019). Metabolic consequences of T-cell costimulation in anticancer immunity. Cancer Immunol Res.

[7] Wu TD, Madireddi S, de Almeida PE (2020). Peripheral T cell expansion predicts tumour infiltration and clinical response. Nature.

[8] Chang CH, Qiu J, O’Sullivan D (2015). Metabolic competition in the tumor microenvironment is a driver of cancer progression. Cell.

[9] Scharping NE, Menk AV, Moreci RS (2016). The tumor microenvironment represses T cell mitochondrial biogenesis to drive intratumoral T cell metabolic insufficiency and dysfunction. Immunity.

[10] Angelin A, Gil-de-Gomez L, Dahiya S (2017). Foxp3 reprograms T cell metabolism to function in low-glucose, high-lactate environments. Cell Metab.

[11] Madden MZ, Rathmell JC (2021). The complex integration of T-cell metabolism and immunotherapy. Cancer Discov.

[12] Wang H, Franco F, Tsui YC (2020). CD36-mediated metabolic adaptation supports regulatory T cell survival and function in tumors. Nat Immunol.

[13] Rao D, Verburg F, Renner K (2021). Metabolic profiles of regulatory T cells in the tumour microenvironment. Cancer Immunol Immunother.

[14] Yost KE, Satpathy AT, Wells DK (2019). Clonal replacement of tumor-specific T cells following PD-1 blockade. Nat Med.

[15] Hiam-Galvez KJ, Allen BM, Spitzer MH (2021). Systemic immunity in cancer. Nat Rev Cancer.

[16] Griffiths JI, Wallet P, Pflieger LT (2020). Circulating immune cell phenotype dynamics reflect the strength of tumor-immune cell interactions in patients during immunotherapy. Proc Natl Acad Sci USA.

[17] Ghiselli F, Milani L (2020). Linking the mitochondrial genotype to phenotype: a complex endeavour. Philos Trans R Soc Lond B Biol Sci.

[18] Stewart JB, Chinnery PF (2021). Extreme heterogeneity of human mitochondrial DNA from organelles to population. Nat Rev Genet.

[19] Shimada K, Crother TR, Karlin J (2012). Oxidized mitochondrial DNA activates the NLRP3 inflammasome during apoptosis. Immunity.

[20] Zhang Q, Raoof M, Chen Y (2010). Circulating mitochondrial DAMPs cause inflammatory responses to injury. Nature.

[21] West AP, Shadel GS (2017). Mitochondrial DNA in innate immune responses and inflammatory pathology. Nat Rev Immunol.

[22] Illumina Human mtDNA Genome Guide. https://emea.support.illumina.com/content/dam/illumina-support/documents/documentation/chemistry_documentation/samplepreps_legacy/human-mtdna-genome-guide-15037958-01.pdf. Accessed 27 Dec 2020.

[23] Anderson S, Bankier AT, Barrell BG (1981). Sequence and organization of the human mitochondrial genome. Nature.

[24] Andrews RM, Kubacka I, Chinnery PF (1999). Reanalysis and revision of the Cambridge reference sequence for human mitochondrial DNA. Nat Genet.

[25] Li H, Durbin R (2009). Fast and accurate short read alignment with Burrows-Wheeler transform. Bioinformatics.

[26] McKenna A, Hanna M, Banks E (2010). The Genome Analysis Toolkit: a MapReduce framework for analyzing next-generation DNA sequencing data. Genome Res.

[27] van Oven M, Kayser M (2009). Updated comprehensive phylogenetic tree of global human mitochondrial DNA variation. Hum Mutat.

[28] Kloss-Brandstätter A, Pacher D, Schönherr S (2011). HaploGrep: a fast and reliable algorithm for automatic classification of mitochondrial DNA haplogroups. Hum Mutat.

[29] Weissensteiner H, Pacher D, Kloss-Brandstätter A (2016). HaploGrep 2: mitochondrial haplogroup classification in the era of high-throughput sequencing. Nucleic Acids Res.

[30] McLaren W, Gil L, Hunt SE (2016). The ensembl variant effect predictor. Genome Biol.

[31] Wang W, Scheffler K, Esbensen Y (2016). Quantification of DNA damage by real-time qPCR. Methods Mol Biol.

[32] Bousquet PA, Meltzer S, Sønstevold L (2019). Markers of mitochondrial metabolism in tumor hypoxia, systemic inflammation, and adverse outcome of rectal cancer. Transl Oncol.

[33] Kristjansson D, Bohlin J, Jugessur A (2021). Matrilineal diversity and population history of Norwegians. Am J Phys Anthropol.

[34] Torroni A, Huoponen K, Francalacci P (1996). Classification of European mtDNAs from an analysis of three European populations. Genetics.

[35] Loogväli EL, Roostalu U, Malyarchuk BA (2004). Disuniting uniformity: a pied cladistic canvas of mtDNA haplogroup H in Eurasia. Mol Biol Evol.

[36] Borst J, Ahrends T, Babala N (2018). CD4^+^ T cell help in cancer immunology and immunotherapy. Nat Rev Immunol.

[37] Previte DM, Martins CP, O’Connor EC (2019). Lymphocyte activation gene-3 maintains mitochondrial and metabolic quiescence in native CD4^+^ cells. Cell Rep.

[38] Ohmura H, Yamaguchi K, Hanamura F (2020). OX40 and LAG3 are associated with better prognosis in advanced gastric cancer patients treated with anti-programmed death-1 antibody. Br J Cancer.

[39] Ahrends T, Spanjaard A, Pilzecker B (2017). CD4^+^ T cell help confers a cytotoxic T cell effector program including coinhibitory receptor downregulation and increased tissue invasiveness. Immunity.

[40] Tsukamoto H, Fujieda K, Senju S (2018). Immune-suppressive effects of interleukin-6 on T-cell-mediated anti-tumor immunity. Cancer Sci.

[41] Kitamura H, Ohno Y, Toyoshima Y (2017). Interleukin-6/STAT3 signaling as a promising target to improve the efficacy of cancer immunotherapy. Cancer Sci.

[42] Jones SA, Jenkins BJ (2018). Recent insights into targeting the IL-6 cytokine family in inflammatory diseases and cancer. Nat Rev Immunol.

[43] Thomsen M, Kersten C, Sorbye H (2016). Interleukin-6 and C-reactive protein as prognostic biomarkers in metastatic colorectal cancer. Oncotarget.

[44] Borst J, Busselaar J, Bosma DMT (2021). Mechanism of action of PD-1 receptor/ligand targeted cancer immunotherapy. Eur J Immunol.

[45] Ruiz-Pesini E, Mishmar D, Brandon M (2004). Effects of purifying and adaptive selection on regional variation in human mtDNA. Science.

[46] Wallace DC (2015). Mitochondrial DNA variation in human radiation and disease. Cell.

[47] Baudouin SV, Saunders D, Tiangyou W (2005). Mitochondrial DNA and survival after sepsis: a prospective study. Lancet.

[48] Jiménez-Sousa MA, Tamayo E, Guzmán-Fulgencio M (2015). Mitochondrial DNA haplogroups are associated with severe sepsis and mortality in patients who underwent major surgery. J Infect.

[49] Guthrie GJK, Roxburgh CSD, Richards CH (2013). Circulating IL-6 concentrations link tumour necrosis and systemic and local inflammatory responses in patients undergoing resection for colorectal cancer. Br J Cancer.

[50] Bray F, Ferlay J, Soerjomataram I (2018). Global cancer statistics 2018: GLOBOCAN estimates of incidence and mortality worldwide for 36 cancers in 185 countries. CA Cancer J Clin.

[51] Bosset JF, Calais G, Mineur L (2014). Fluorouracil-based adjuvant chemotherapy after preoperative chemoradiotherapy in rectal cancer: long-term results of the EORTC 22921 randomised study. Lancet Oncol.

[52] Rödel C, Graeven U, Fietkau R (2015). Oxaliplatin added to fluorouracil-based preoperative chemoradiotherapy and postoperative chemotherapy of locally advanced rectal cancer (the German CAO/ARO/AIO-04 study): final results of the multicentre, open-label, randomised, phase 3 trial. Lancet Oncol.

