# Supplementary material for: The mitochondrial DNA constitution shaping T-cell immunity in patients with rectal cancer at high risk of metastatic progression
Source: Clin Transl Oncol. 2021 Dec 27;24(6):1157–67. doi: 10.1007/s12094-021-02756-w (PMC9107448; doi:10.1007/s12094-021-02756-w)
Supplement: Supplementary file 1 — Supplementary file1 (DOCX 750 kb) [file 12094_2021_2756_MOESM1_ESM.docx]

**Supplementary Information**

**The mitochondrial DNA constitution shaping T-cell immunity in patients with rectal cancer at high risk of metastatic progression**

***Clinical and Translational Oncology***

P. A. Bousquet^1^, S. Meltzer^1^, A. J. Fuglestad^1,2,3^, T. Lüders^4,2^, Y. Esbensen^4,2^, H. V. Juul^5^, C. Johansen^1^, L. G. Lyckander^6^, T. Bjørnetrø^1^, E. M. Inderberg^5^, C. Kersten^3,1^, K. R. Redalen^7,1^, A. H. Ree^1,2,^*

^1^ Department of Oncology, Akershus University Hospital, Lørenskog, Norway

^2^ Institute of Clinical Medicine, University of Oslo, Oslo, Norway

^3^ Centre for Cancer Treatment, Sørlandet Hospital, Kristiansand, Norway

^4^ Department of Clinical Molecular Biology, Akershus University Hospital, Lørenskog, Norway

^5^ Department of Cellular Therapy, Oslo University Hospital, Oslo, Norway

^6^ Department of Pathology, Akershus University Hospital, Lørenskog, Norway

^7^ Department of Physics, Norwegian University of Science and Technology, Trondheim, Norway

* Corresponding author: [a.h.ree@medisin.uio.no](mailto:a.h.ree@medisin.uio.no)

**Supplementary methods**

**Patients and procedures**

The study (ClinicalTrials.gov NCT01816607) was conducted at Akershus University Hospital (Lørenskog, Norway) with a catchment area that covers a tenth of the Norwegian population and reflects essential demographics (age groups, socio-economic distribution and ethnic composition) of the nation. Patients were enrolled according to unselected recruitment. Eligible patients were 18 years or older with no prior radiation therapy for pelvic neoplasia. They had histologically verified rectal adenocarcinoma that was considered high risk by magnetic resonance imaging: T2 cases that presented tumour threatening the anal canal muscles, T3 cases that had mesorectal fascia margin of less than 2 mm, T4 cases (organ-infiltrating tumour) or cases that had involved pelvic cavity lymph nodes (N1-2 disease). The absence of metastatic disease at the time of diagnosis was established on computed tomography scans of the thoracic and abdominal cavities. Tumour biopsies were tested for microsatellite status on the Idylla platform (Biocartis NV), using 10-µm sections of 25-300 mm^2^ tissue with at least 20% tumour cells, as determined by an experienced specialist in gastrointestinal pathology, which were prepared and analysed according to the manufacturer’s instructions.

The 44 patients reported here were enrolled between 28 October 2013 and 14 November 2017 and treated according to the prevailing national guidelines with neoadjuvant radiation therapy and radical pelvic surgery. For 41 patients, the radiation to the tumour bed and regional lymph nodes consisted of long-course radiation delivered in 25 fractions over 5 weeks to a total dose of 50 Gy with concomitant capecitabine (825 mg/m^2^ twice daily on days of radiation). Because of relevant comorbidity, two patients had only the long-course radiation. One patient received a short-course regimen consisting of radiation delivered in 5 fractions over 1 week to a total dose of 25 Gy because of frailty. Radical excision of the residual tumour within its entire extension was performed at a median of 8.0 (minimum 5.0, maximum 13) weeks after completion of the neoadjuvant therapy. Of note, two patients refused surgery due to personal opinions. Patients did not proceed to post-operative treatment.

The resected tumour specimens were histologically evaluated by the specialist in gastrointestinal pathology for local treatment response (ypTN stage) and tumour regression grade. In this, grade 1 represents <5% residual tumour cells (near-complete or complete response), grade 2 represents 5-50% residual tumour cells and grade 3 represents >50% residual tumour cells [1]. Patients were followed with regular clinical and computed tomography examinations for five years after the completion of the multimodal treatment to record metastatic events in distant organs beyond the pelvic cavity (defining distant metastasis-free survival), with median follow-up of 38 (minimum 2, maximum 60) months at censoring on 2 January 2020.

**Preparation of patient samples**

Whole blood (WB) samples were collected by venipuncture in PAXgene RNA tubes (PreAnalytiX) at the time of diagnosis, for DNA extraction undertaken after median 45 (minimum 16, maximum 66) months of storage at –80°C. Peripheral blood mononuclear cell (PBMC) specimens were prepared from 6-8 mL of the WB by centrifugation with a horizontal rotor centrifuge at 1500×*g* for 20 minutes. The buffy coat layer was transferred to a fresh 15 mL tube, resuspended and washed twice in phosphate-buffered saline with centrifugations at 300×*g* for 15 and 10 minutes. The mononuclear cells were thereafter resuspended in RPMI-1640 medium supplemented with dimethyl sulfoxide (9:1) and immediately frozen in –150°C. For serum preparation, WB was drawn in plain tubes with no additives for centrifugation to separate serum, which was left on ice for no more than 1 hour before storage at –80°C.

Prior to DNA extraction, 150 µL of thawed PBMC preparations or PAXgene samples were transferred to microcentrifuge tubes and centrifuged at 5000×*g* for 10 minutes before the supernatants were carefully removed. Serum samples were centrifuged one more time (at 2000×*g* for 15 minutes) following thawing and 50 µL were used. Total DNA was extracted using the DNeasy Blood & Tissue Kit (Qiagen), according to the manufacturer’s instructions, and the DNA was subsequently quantified using the Qubit fluorometer 2.0 (Thermo Fisher Scientific) in combination with the Qubit dsDNA HS Assay Kit (Thermo Fisher Scientific).

**Healthy blood donor samples**

The procedures are described in the main text of the article.

#### Library preparation and sequencing

#### The procedures are described in the main text of the article.

**Sequence analysis**

The procedures are described in the main text of the article.

**High-dimensional single-cell mass cytometry**

PBMC preparations available from 32 of the rectal cancer patients and 10 healthy blood donors were thawed and plated in 24-well plates at a concentration of 0.5-2.0×10^6^ cells/mL. The protein transport inhibitors BP GolgiStop and BD GolgiPlug (BD Biosciences) were added to all wells at 1:1000 dilutions. PBMC samples containing >2×10^6^ cells were split in two and one of the halves was stimulated with 20 ng/mL phorbol myristate acetate and 1 µM Ionomycin (both Sigma-Aldrich) for expression of markers of cytotoxicity and cytokine production (the intracellular antibody panel; below). Cells were incubated at 37°C in 5% CO_2_ for 6 hours and then kept at 4°C overnight. Cells were subsequently washed and resuspended in the Maxpar Cell Staining Buffer (Fluidigm) and stained with the Cell-ID Cisplatin solution (Fludigm) for 5 minutes, before washing and staining with the extracellular antibodies (the extracellular antibody panel; below) for 30 minutes. Next, the samples were fixed with 1.6% paraformaldehyde and permeabilized in 99% methanol (Sigma-Aldrich) at –80°C to be stored for maximum 4 weeks. Before the final fixation and permeabilization, the cell samples that had been stimulated for analysis of intracellular proteins had the methanol removed and were additionally stained with the respective antibodies for 20 minutes. Finally, all samples were incubated with the iridium-containing Cell-ID Intercalator-Ir cell tracker solution (Fluidigm) for 20 minutes and run on a CyTOF 2 Mass Cytometer (Fluidigm) at the Flow Cytometry Core Facility at Oslo University Hospital. The data were normalised using EQ Four Element Calibration Beads (Fluidigm).

**Mass cytometry data analysis**

The procedures are described in the main text of the article.

**Quantification of mtDNA damage**

The assay relies on the ability of a structural modification within a 4-base site (TCGA, which did not exhibit polymorphisms in any of the patient samples) on the template DNA to inhibit restriction enzyme cleavage [2]. As we have published previously [3], following the serum DNA isolation, a sequence flanking a TaqI restriction enzyme site in the gene encoding the 12S ribosomal subunit (*MT-RNR1*) was amplified using the forward (5’-AAACTGCTCGCCAGAACACT-3’) and reverse (5’-CATGGGCTACACCTTGACCT-3’) primers in the absence and presence of the enzyme. For the quantitative digital PCR analysis, samples were partitioned by the QX200 Droplet Generator (Bio-Rad Laboratories) and analysed with the QX200 Droplet Reader (Bio-Rad Laboratories). Data were given as the percentage of non-digested (nd) mtDNA [(mtDNA^TaqI^ copies/μL – mtDNA^nd^ copies/μL) × 100].

**Analysis of serum cytokines**

The procedures are described in the main text of the article.

**Statistical analysis**

All details are given in the main text of the article.

**The intracellular antibody panel**

|  | Antigen | Tag | Clone | Supplier | Catalog no. |
| --- | --- | --- | --- | --- | --- |
| 1 | IL-5 | 143Nd | TRFK5 | Fluidigm | 314003B |
| 2 | IL-4 | 144Nd | MP4-25D2 | Fluidigm | 3144010B |
| 3 | CD107a (LAMP-1) | 151Eu | H4A3 | Fluidigm | 3151002B |
| 4 | TNF-α | 152Sm | Mab11 | Fluidigm | 3152002B |
| 5 | IL-6 | 156Gd | MQ2-13A5 | Fluidigm | 3156011B |
| 6 | IL-2 | 158Gd | MQ1-17H12 | Fluidigm | 3159008B |
| 7 | GM-CSF | 159Tb | BVD2-21C11 | Fluidigm | 3159008B |
| 8 | MIP1-β | 160Gd | D21-1351 | Fluidigm | 3160013B |
| 9 | CD69 | 162Dy | FN50 | Fluidigm | 3162001B |
| 10 | IL-17A | 164Dy | N49-653 | Fluidigm | 3164002B |
| 11 | IL-17F | 166Er | SHLR17 | Fluidigm | 3166010B |
| 12 | IFN-γ | 168Er | B27 | Fluidigm | 3168005B |
| 13 | Granzyme B | 171Yb | GB11 | Fluidigm | 3171002B |
| 14 | Perforin | 175Lu | B-D48 | Fluidigm | 3175004B |
| 15 | CD4 | 145Nd | RPA-T4 | Fluidigm | 3145001B |
| 16 | CD8a | 146Nd | RPA-T8 | Fluidigm | 3146001B |
| 17 | CD3 | 170Er | UCHT1 | Fluidigm | 3170001B |
| 18 | CD19 | 165Ho | HIB19 | Fluidigm | 3165025B |
| 19 | CD38 | 167Er | HIT2 | Fluidigm | 3167001B |
| 20 | CD56 | 176Yb | NCAM16.2 | Fluidigm | 3176008B |
| 21 | CD16 (FcgRIII) | 209Bi | 3G8 | Fluidigm | 3209002B |
| 22 | Ki-67 | 161Dy | B56 | Fluidigm | 3161007B |
| 23 | LAG-3 (CD223) | 150Nd | 874501 | Fluidigm | 3150016B |
| 24 | TIM-3 | 153Eu | F38-2E2 | Fluidigm | 3153008B |
| 25 | TIGIT | 154Sm | MBSA43 | Fluidigm | 3154016B |
| 26 | CD279 (PD-1) | 155Gd | EH12.2H7 | Fluidigm | 3155009B |

**The extracellular antibody panel**

|  | Antigen | Tag | Clone | Supplier | Catalog no. |
| --- | --- | --- | --- | --- | --- |
| 1 | CD45 | 89Y | HI30 | Fluidigm | 3089003B |
| 2 | CD196 (CCR6) | 141Pr | G034E3 | Fluidigm | 3141003A |
| 3 | CD19 | 142Nd | HIB19 | Fluidigm | 3142001B |
| 4 | CD45RA | 143Nd | HI100 | Fluidigm | 3143006B |
| 5 | CD11b | 144Nd | GHI/61 | Fluidigm | 3144001B |
| 6 | CD4 | 145Nd | RPA-T4 | Fluidigm | 3145001B |
| 7 | IgD | 146Nd | 1A6-2 | Fluidigm | 3146005B |
| 8 | CD20 | 147Sm | 2H7 | Fluidigm | 3147001B |
| 9 | CD274 (PD-L1) | 148Nd | 29E.2A3 | Fluidigm | 3148017B |
| 10 | CD25 | 149Sm | 2A3 | Fluidigm | 3149010B |
| 11 | LAG-3 (CD223) | 150Nd | 874501 | Fluidigm | 3150016B |
| 12 | CD278 (ICOS) | 151Eu | DX29 | Fluidigm | 3151008B |
| 13 | CD21 | 152Sm | BL13 | Fluidigm | 3152010B |
| 14 | TIM-3 | 153Eu | F38-2E2 | Fluidigm | 3153008B |
| 15 | TIGIT | 154Sm | MBSA43 | Fluidigm | 3154016B |
| 16 | CD27 | 155Gd | L128 | Fluidigm | 3155001B |
| 17 | CD14 | 156Gd | HCD14 | Fluidigm | 3156019B |
| 18 | CD137 | 158Gd | 4B4-1 | Fluidigm | 3158013B |
| 19 | CD197 (CCR7) | 159Tb | G043H7 | Fluidigm | 3159003A |
| 20 | CD28 | 160Gd | CD28.2 | Fluidigm | 3160003B |
| 21 | CTLA-4 | 161Dy | 14D3 | Fluidigm | 3161004B |
| 22 | CD183 (CXCR3) | 163Dy | G025H7 | Fluidigm | 3163004B |
| 23 | CD161 | 164Dy | HP-3G10 | Fluidigm | 3164009B |
| 24 | CD127 | 165Ho | A019D5 | Fluidigm | 3165008B |
| 25 | NKG2D (CD314) | 166Er | ON72 | Fluidigm | 3166016B |
| 26 | CD38 | 167Er | HIT2 | Fluidigm | 3167001B |
| 27 | CD8a | 168Er | SK1 | Fluidigm | 3168002B |
| 18 | CD33 | 169Tm | WM53 | Fluidigm | 3169010B |
| 19 | CD3 | 170Er | UCHT1 | Fluidigm | 3170001B |
| 30 | CD185 (CXCR5) | 171Yb | 51505 | Fluidigm | 3171006B |
| 31 | CD273 (PD-L2) | 172Yb | 24F.10C12 | Fluidigm | 3172014B |
| 32 | CD184 (CXCR4) | 173Yb | 12G5 | Fluidigm | 3173001B |
| 33 | HLA-DR | 174Yb | L243 | Fluidigm | 3174001B |
| 34 | CD279 (PD-1) | 175Lu | EH12.2H7 | Fluidigm | 3175008B |
| 35 | CD56 | 176Yb | NCAM16.2 | Fluidigm | 3176008B |
| 36 | CD16 (FcgRIII) | 209Bi | 3G8 | Fluidigm | 3209002B |

**Supplementary references**

1. Bateman AC, Jaynes E, Bateman AR. Rectal cancer staging post neoadjuvant therapy – how should the changes be assessed? Histopathology. 2009;54:713–21.

2. Wang W, Scheffler K, Esbensen Y, Eide L. Quantification of DNA damage by real-time qPCR. Methods Mol Biol. 2016;1351:27–32.

3. Bousquet PA, Meltzer S, Sønstevold L, Esbensen Y, Dueland S, Flatmark K, Sitter B, Bathen TF, Seierstad T, Redalen KR, Eide L, Ree AH. Markers of mitochondrial metabolism in tumor hypoxia, systemic inflammation, and adverse outcome of rectal cancer. Transl Oncol. 2019;12:76–83.

**Fig. S1.** Scatter graphs for significant correlations


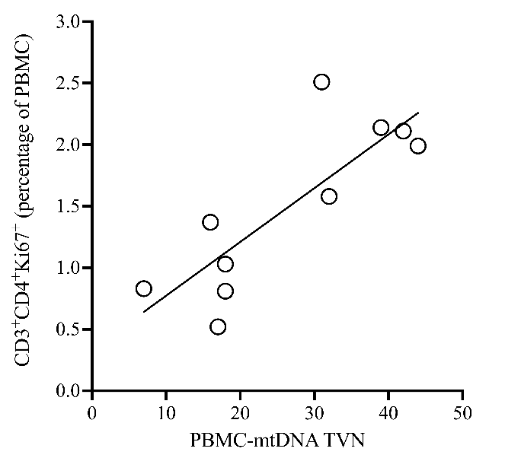

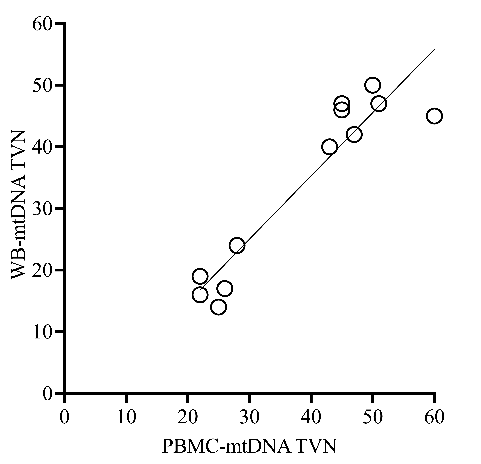


*rho* = 0.687, *p* = 0.033;

by Spearman correlation test

*r* = 0.939, *p* < 0.001;

by Pearson correlation test


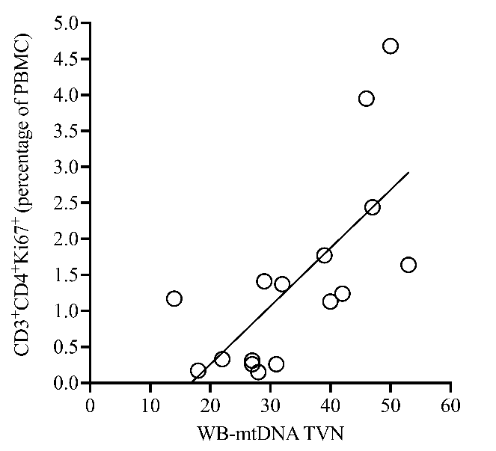

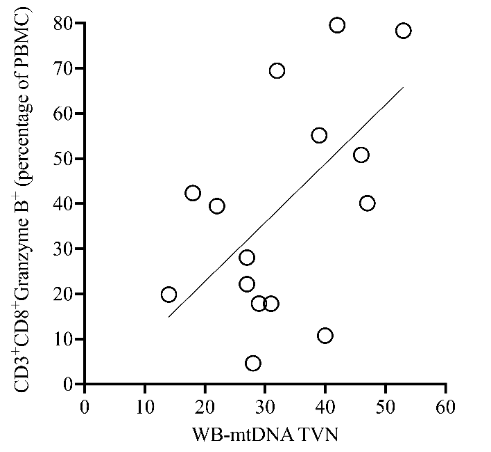


*rho* = 0.534, *p* = 0.033;

by Spearman correlation test

*rho* = 0.734, *p* = 0.001;

by Spearman correlation test


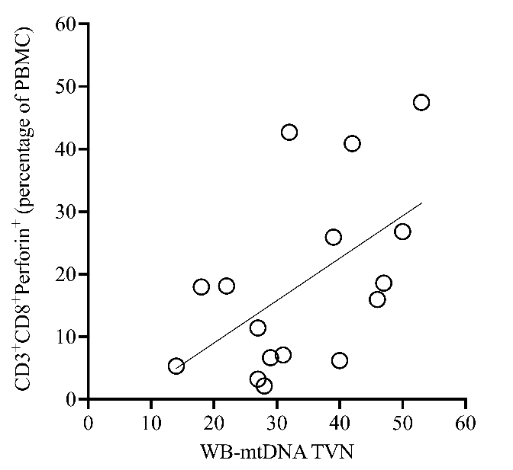

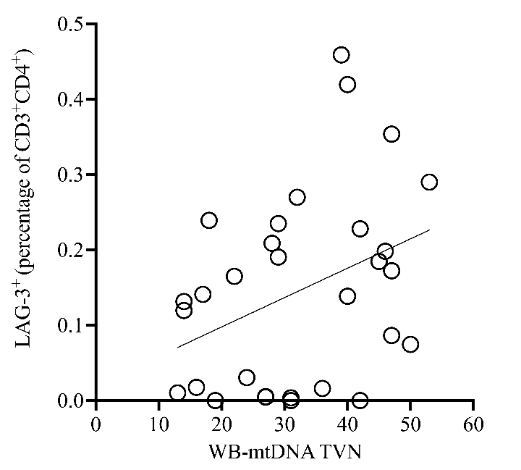

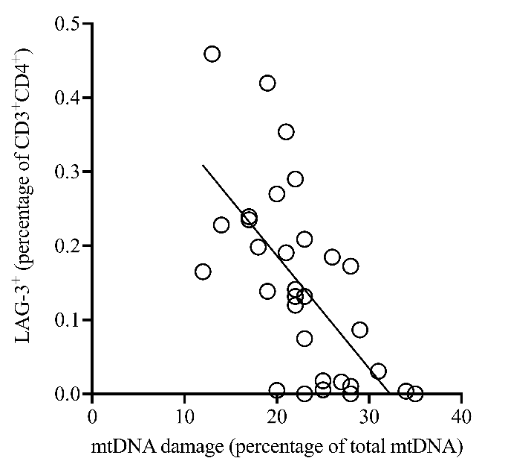

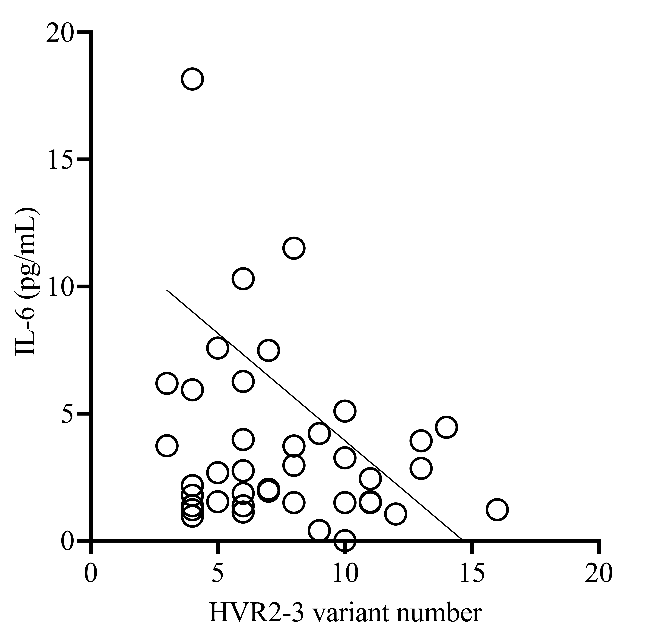


*r* = 0.378, *p* = 0.033;

by Pearson correlation test

*rho* = 0.590, *p* = 0.016;

by Spearman correlation test

**Table S1** Patient and tumour characteristics

*rho* = –0.703, *p* < 0.001;

by Spearman correlation test

*rho* = –0.347, *p* = 0.025;

by Spearman correlation test

|  |  | *n* (%) | Median  (min, max) | TVN,  mean (SD) | | *r* | *p*^a^ |
| --- | --- | --- | --- | --- | --- | --- | --- |
| Age, years |  | 44 (100) | 63 (41, 79) | 31.0 (11.4) | –0.069 | | 0.66 |
| Sex | Male | 32 (72.7) |  | 30.6 (10.5) |  | | 0.73 |
|  | Female | 12 (27.3) |  | 32.0 (13.9) |  | |  |
| Body mass index, kg/m^2^ |  | 44 (100) | 24 (17, 35) | 31.0 (11.4) | –0.036 | | 0.82 |
| Metformin use | No | 41 (93.2) |  | 31.2 (11.4) |  | | 0.61 |
|  | Yes | 3 (6.8) |  | 27.7 (12.5) |  | |  |
| Tumour microsatellite status | Stable | 44 (100) |  | 31.0 (11.4) |  | |  |
|  | Instable | 0 |  |  |  | |  |
| T stage | 2 | 4 (9.1) |  | 37.5 (7.9) |  | | 0.50 |
|  | 3 | 21 (47.7) |  | 30.2 (11.8) |  | |  |
|  | 4 | 19 (43.2) |  | 30.5 (11.5) |  | |  |
| N stage | 0 | 18 (40.9) |  | 29.0 (10.1) |  | | 0.56 |
|  | 1 | 16 (36.4) |  | 33.3 (12.6) |  | |  |
|  | 2 | 10 (22.7) |  | 31.0 (12.1) |  | |  |
| Neoadjuvant therapy | CRT | 41 (93.2) |  | 31.4 (11.3) |  | | 0.42 |
|  | LCRT | 2 (4.5) |  | 30.5 (16.3) |  | |  |
|  | SCRT | 1 (2.3) |  | 16 |  | |  |
| ypT stage | 0 | 8 (18.2) |  | 34.3 (10.8) |  | | 0.36 |
|  | 1 | 4 (9.1) |  | 33.5 (10.3) |  | |  |
|  | 2 | 5 (11.4) |  | 33.6 (13.3) |  | |  |
|  | 3 | 23 (52.3) |  | 27.4 (11.4) |  | |  |
|  | 4 | 2 (4.5) |  | 41.0 (12.7) |  | |  |
|  | ND^b^ | 2 (4.5) |  | 37.5 (2.1) |  | |  |
| ypN stage | 0 | 28 (63.6) |  | 29.1 (11.3) |  | | 0.33 |
|  | 1 | 9 (20.5) |  | 36.3 (11.1) |  | |  |
|  | 2 | 5 (11.4) |  | 29.6 (13.2) |  | |  |
|  | ND^b^ | 2 (4.5) |  | 37.5 (2.1) |  | |  |
| TRG | 1 | 19 (43.2) |  | 33.2 (11.0) |  | | 0.22 |
|  | 2 | 18 (40.9) |  | 30.5 (11.8) |  | |  |
|  | 3 | 5 (11.4) |  | 22.0 (10.3) |  | |  |
|  | ND^b^ | 2 (4.5) |  | 37.5 (2.1) |  | |  |

^a^By Pearson correlation test, Student’s *t*-test or one-way analysis of variance, as appropriate

^b^Patients who rejected surgery after the neoadjuvant therapy due to personal opinion

Abbreviations: CRT, chemoradiotherapy (radiation delivered in 25 fractions over 5 weeks to a total dose of 50 Gy with concomitant capecitabine); LCRT, long-course radiation therapy (delivered in 25 fractions over 5 weeks to a total dose of 50 Gy); max, maximum; min, minimum; N, node; ND, not determined (TVN value not included in statistical calculation); SCRT, short-course radiation therapy (delivered in 5 fractions over 1 week to a total dose of 25 Gy); SD, standard deviation; T, tumour; TRG, histologic tumour regression grade [from near-complete or complete (1) to none (3)]; TVN, mitochondrial DNA total variant number; yp, histologic stage after neoadjuvant therapy

**Fig. S2** Whole blood (WB) and peripheral blood mononuclear cells (PBMC) mitochondrial DNA (mtDNA) variant numbers in 12 high-risk rectal cancer patients


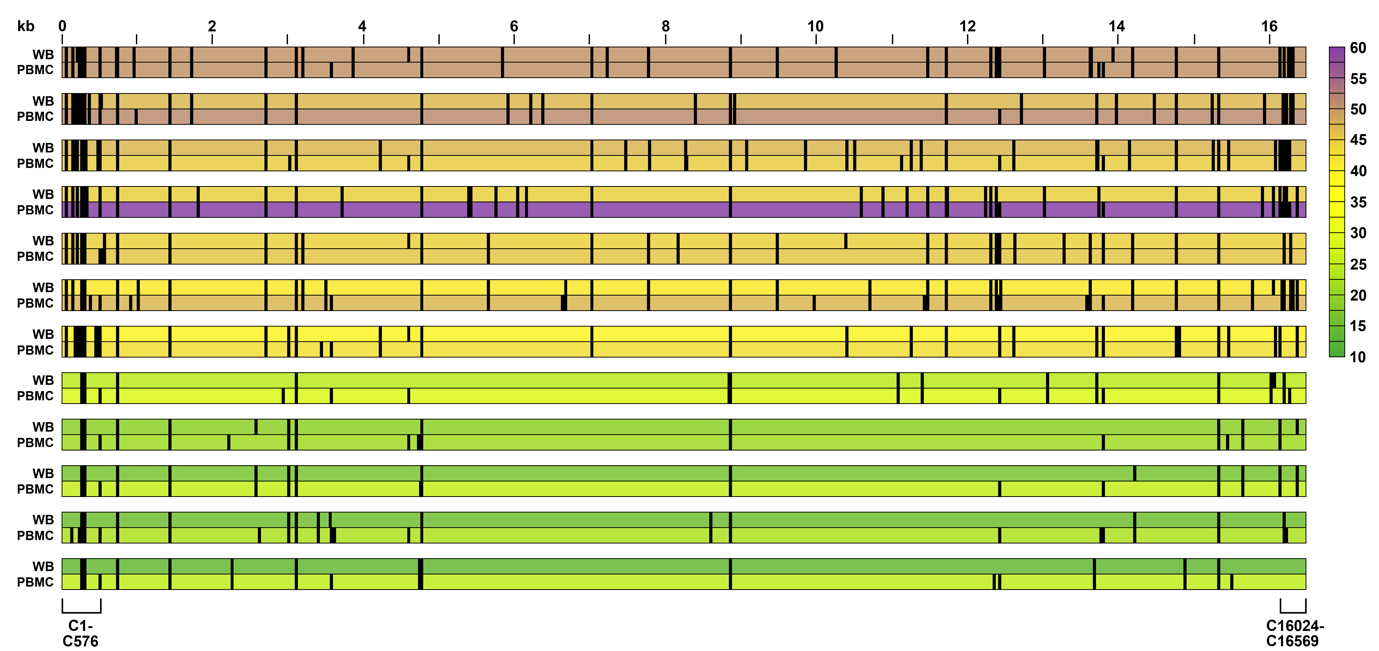


Each pair of rows represents one individual patient. The positions across the entire ~16.6 kilobase (kb) mitochondrial genome are indicated on the top of the panel. The control (C) region’s base sites 1-576 and 16024-16569 are specified below the panel. Each vertical line indicates the position of an mtDNA base with at least one variant other than the reference, as called by the curated reference genome (mitomap.org). The total number of base variants in each case is indicated by colour (scale to the right). While the PBMC median number of mtDNA variants was 44 (minimum 22, maximum 60), the WB median number was 41 (minimum 14, maximum 50).

**Table S2** Associations between WB-mtDNA TVN, patient- or tumour characteristics and DMFS

|  | *n*^a^ | HR (95% CI) | *p* |
| --- | --- | --- | --- |
| WB-mtDNA TVN | 42 | 0.929 (0.871-0.991) | 0.026 |
| Variables not remaining in the equation | | | |
| Age | 42 |  | 0.143 |
| Sex | 42 |  | 0.915 |
| Body mass index | 42 |  | 0.133 |
| Metformin use | 42 |  | 0.543 |
| T stage | 42 |  | 0.345 |
| N stage | 42 |  | 0.367 |
| ypT stage | 42 |  | 0.079 |
| ypN stage | 42 |  | 0.073 |
| TRG | 42 |  | 0.150 |

HR below 1 indicates favourable DMFS with higher WB-mtDNA TVN, from a multivariable Cox proportional hazard model with forward conditional selection

^a^Excludes the two patients who rejected surgery after the neoadjuvant therapy

Abbreviations: CI, confidence interval; DMFS, distant metastasis-free survival; HR, hazard ratio; N, node; T, tumour; TRG, histologic tumour regression grade [from near-complete or complete (1) to none (3)]; TVN, total variant number; WB-mtDNA, whole blood mitochondrial DNA; yp, histologic stage after neoadjuvant therapy

**Fig. S3** Polymorphisms of the 3105 site of whole blood mitochondrial DNA in 44 high-risk rectal cancer patients


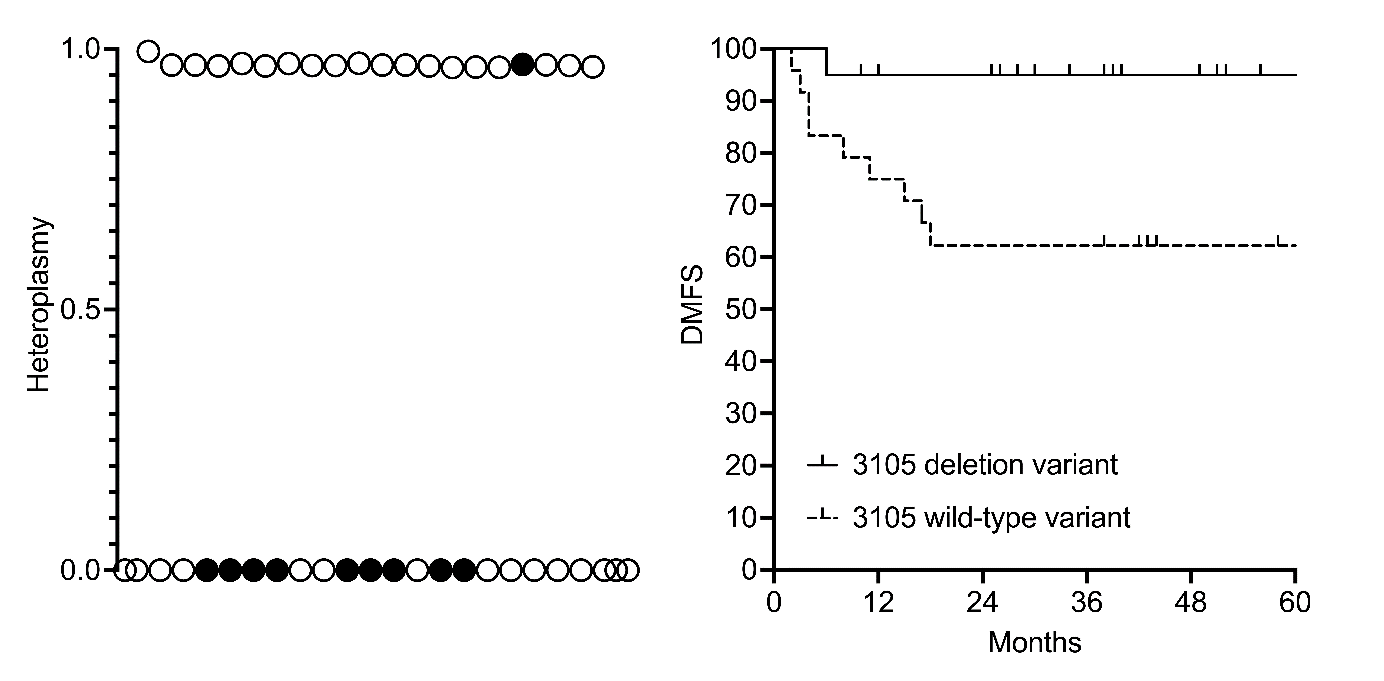


Left panel: Cases with either the wild-type site (heteroplasmy of 0) or a highly heteroplasmic AC>A variant; open circles: cases without progression; closed circles: cases with metastatic progression. Right panel: Distant metastasis-free survival (DMFS) in the two patient groups (*p* = 0.014; by log-rank test).

**Fig. S4** Mitochondrial DNA (mtDNA) variant number in peripheral blood mononuclear cells from 10 healthy individuals (blood donors)


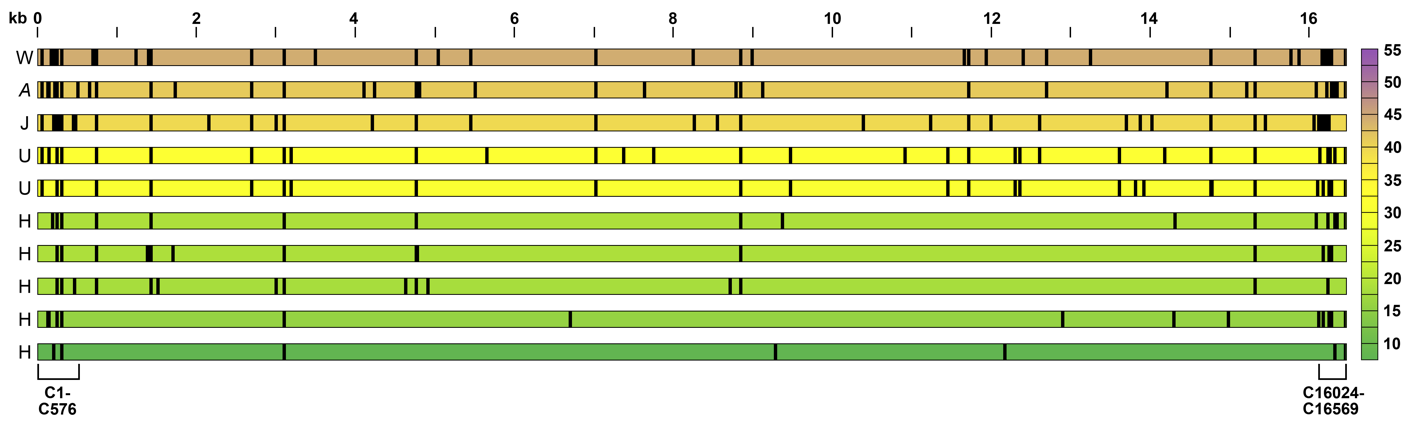


Each row represents one individual person with the haplogroup shown to the left (the non-European A haplogroup in italic). The positions across the entire ~16.6 kilobase (kb) mitochondrial genome are indicated on the top of the panel. The control (C) region’s base sites 1-576 and 16024-16569 are specified below the panel. Each vertical line indicates the position of an mtDNA base with at least one variant other than the reference, as called by the curated reference genome (mitomap.org). The total number of base variants in each case is indicated by colour (scale to the right).

**Fig. S5** viSNE maps of circulating immune cells in a study patient without metastatic failure (left panels) and the frequencies of the cell populations in 32 analysed patient samples (right panels)


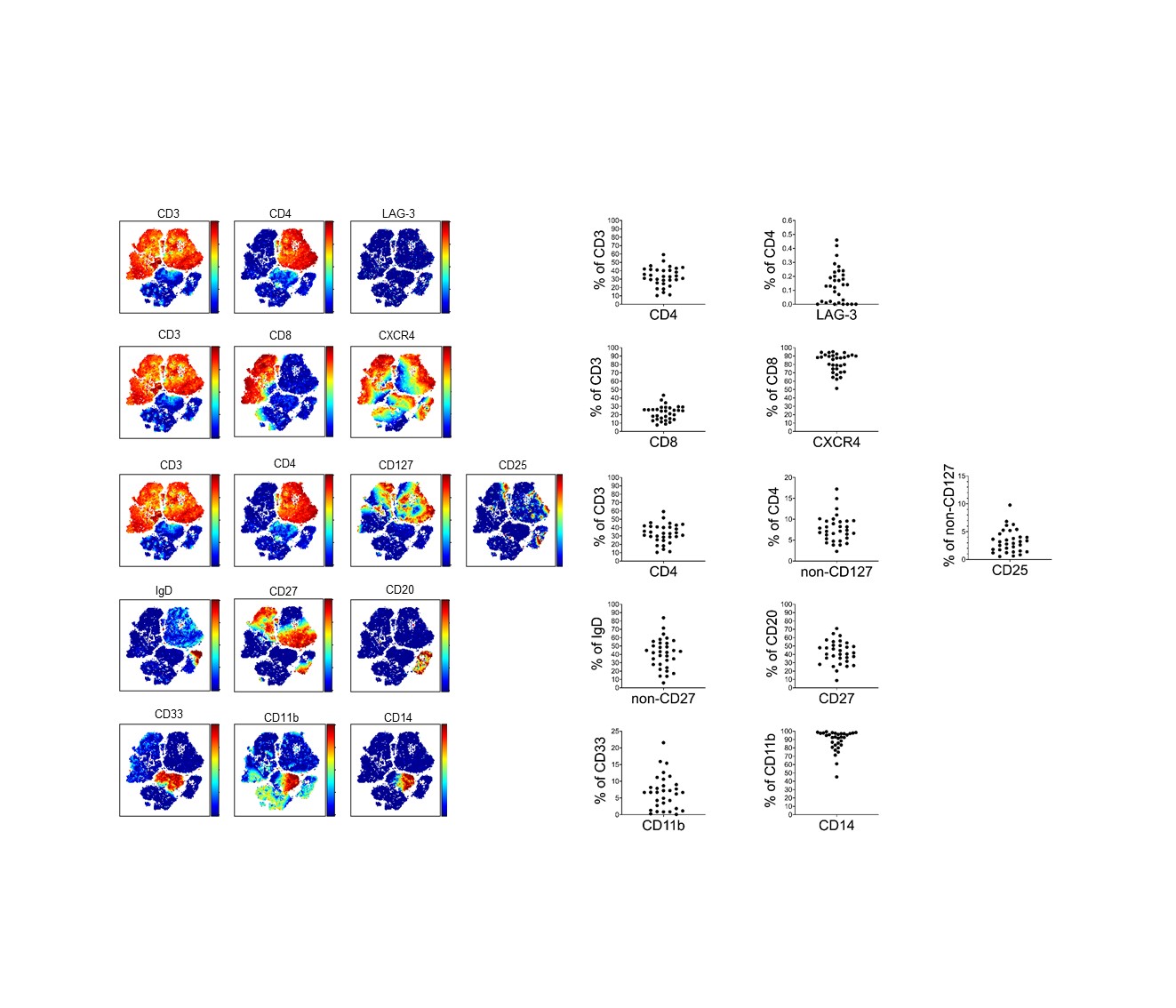


Cell populations were visualised by viSNE mapping based on the t-Distributed Stochastic Neighbor Embedding clustering algorithm. From the top: the lymphocyte activation gene (LAG)-3 population from which new helper T cells are recruited (row 1), tumour-targeting effector T cells (row 2), immune-suppressive regulatory T cells (row 3), naïve and memory B cells (row 4) and monocytes (row 5). Expression levels of the various immune cell antigens are indicated by colours, from high (red) to low (blue), in each map (scale to the right).

**Table S3** Correlations between mitochondrial DNA variant numbers in whole blood and serum interleukin-6

|  | *n*^a^ | *rho* | *p* |
| --- | --- | --- | --- |
| TVN | 42 | –0.215 | 0.172 |
| TVN without haplogroup-specific variants | 42 | –0.069 | 0.664 |
| TVN without AV | 42 | –0.272 | 0.081 |
| CRVN | 42 | –0.275 | 0.078 |
| CRVN without AV | 42 | –0.212 | 0.178 |
| HVR1 (C16024-C16569) variant number | 42 | –0.058 | 0.716 |
| HVR1 variant number without AV | 42 | –0.014 | 0.928 |
| HVR2-3 (C1-C576) variant number | 42 | –0.347 | 0.025 |
| HVR2-3 variant number without AV | 42 | –0.414 | 0.006 |
| Total AV number | 42 | –0.052 | 0.745 |

Calculated by Spearman correlation test

^a^Excludes two patients who did not have serum interleukin-6 measured

Abbreviations: AV, ancestral variants; C, control region; CRVN, coding region variant number; HVR, hypervariable region (the C region’s base sites within the HVRs are given in brackets); TVN, total variant number
